# Age-Dependent Microglial Response to Systemic Infection

**DOI:** 10.3390/cells10051037

**Published:** 2021-04-28

**Authors:** Brianna Cyr, Juan Pablo de Rivero Vaccari

**Affiliations:** 1 Department of Neurological Surgery and The Miami Project to Cure Paralysis, University of Miami Miller School of Medicine, Miami, FL 33136, USA; bxc205@miami.edu; 2 Center for Cognitive Neuroscience and Aging, University of Miami Miller School of Medicine, Miami, FL 33136, USA

**Keywords:** microglia, aging, inflammation, sepsis, infection

## Abstract

Inflammation is part of the aging process, and the inflammatory innate immune response is more exacerbated in older individuals when compared to younger individuals. Similarly, there is a difference in the response to systemic infection that varies with age. In a recent article by Hoogland et al., the authors studied the microglial response to systemic infection in young (2 months) and middle-aged mice (13–14 months) that were challenged with live *Escherichia coli* to investigate whether the pro- and anti-inflammatory responses mounted by microglia after systemic infection varies with age. Here, we comment on this study and its implications on how inflammation in the brain varies with age.

Historically, the central nervous system (CNS) has usually been seen as immune privileged [1], where the brain and spinal cord are protected from pathogens and infiltrating immune cells. This immune privileged concept is due to the lack of professional antigen-presenting cells in the brain parenchyma, the lack of lymphatic drainage of CNS antigens, and the low expression of major histocompatibility complex (MHC) class Ⅰ and Ⅱ. Together, this creates an environment that limits the ability of the CNS to mount an immune response to antigens originating in the CNS [1]. However, lymphatic drainage has been described in the meninges, at the level of the dura matter, which allow macromolecules and immune cells to exit the CNS through the deep cervical lymph nodes [2]. Therefore, there is evidence for the flow of molecules such as amyloid-β [3] and immune cells between the CNS and the periphery.

In addition, T-cells are important white blood cells of the adaptive innate immune response in the periphery. However, in the brain, T-cells have also been shown to contribute to learning and memory [4], as well as aging [5], suggesting that the presence of immune cells in the brain can be linked to normal physiology. In addition, microglia are the resident macrophages of the CNS. As such, microglia are involved in phagocytosis and the modulation of the pro- and anti-inflammatory response within the CNS. Further supporting the view that the CNS is capable of mounting an immune response that is mediated by other cells besides microglia is the ability of CNS cells such as neurons and oligodendrocytes to mount an innate immune response through constitutively present inflammasomes [6]. Inflammasomes are multiprotein complexes involved in the activation of caspase-1 and the processing of the pro-inflammatory cytokines IL-1β and IL-18, perpetuating the inflammatory signal [7]. Therefore, even though neurons and oligodendrocytes are not classically seen as professional immune cells in the CNS, they are still capable of mounting an immune response [8,9]. Perhaps this characteristic of CNS cells, such as neurons, provides them with the ability to alert professional immune cells, like microglia and infiltrating immune cells, through the release of cytokines, to protect the CNS following damage or infections since neurons are unable to act as antigen-presenting cells. Therefore, the CNS is more capable of mounting an immune response than is normally thought. Furthermore, diseases affecting the CNS [10], as well as CNS injuries [11] and aging [12], bridge the blood brain barrier (BBB), allowing for the trafficking of immune cells between the CNS and the periphery in the context of aging, trauma, or disease.

Similarly, a variety of recent findings highlight the systemic effects of CNS problems, such as pulmonary [13,14] or gastrointestinal effects [15] resulting from CNS trauma. In addition, there are also inflammatory stimuli that originate in the periphery that have an effect on the CNS [16]. For instance, recent evidence implicates the gut microbiome as a contributor to neurodegenerative diseases such as Alzheimer’s disease [17] and Parkinson’s disease [18]. Therefore, there is a crosstalk between the CNS and the periphery that makes the brain less immune privileged than one normally thinks.

Importantly, the inflammatory response that occurs systemically and in the brain is exacerbated as a result of aging [19]. The underlying inflammation, which is actually heightened with age, is believed to be a precursor to neurodegenerative diseases like Alzheimer’s disease [20]. Moreover, in diseases in which inflammation plays a critical role, the aging population seems to be more vulnerable [16]. In addition, male and females tend to be affected differently in a variety of diseases. This is probably in part because the inflammatory response in males and females is also different [21]. Therefore, there is a need to better understand how the inflammatory response varies at different ages as well as how it differs between men and women.

In a previous study by Hoogland et al., the authors studied the effects of a systemic infection with live bacteria on the CNS [22] in which the immune response mounted by microglia was more exacerbated in middle-aged infected mice when compared to young infected mice. Previous research has shown that systemic infection can affect the CNS; studies have shown evidence of cognitive decline following systemic inflammation [23,24,25]. Cognitive decline seems to occur more frequently in the aged population after infection, likely due to the microglial priming that occurs with age [25,26]. Primed microglia have a heightened pro-inflammatory gene profile [26,27,28] and a decrease in neuroprotective factors [28,29], which causes an exaggerated response to stimuli. Based on this evidence, the authors hypothesized that systemic infection would produce a greater pro-inflammatory response in the brain of aged mice, and that this heightened response occurs because aged mice are lacking in the expression of microglial-specific anti-inflammatory genes.

There is a debate over the relevance of lipopolysaccharide (LPS) as a stimulus to mimic sepsis in rodent models. Accordingly, LPS as a systemic challenge induces a greater neuroinflammatory response and microglial activation than a more clinically relevant approach using a systemic challenge with live or heat-killed bacteria [30]. In a study by Hoogland et al., the authors used an animal model in which young adult mice were systemically challenged with live *E. coli* to more closely mimic the clinical setting of sepsis [31]. Accordingly, this model consists of administering a lethal dose of *E. coli* to mice, followed by treatment with ceftriaxone, an antimicrobial drug, 12 and 24 h after *E. coli* injection.

In their recent publication, Hoogland et al. use this novel model to compare the microglial response, inflammatory marker expression, and inhibitory regulators of microglial activation in the brains of young (2 months old) and middle-aged (13 to 14 months old) infected mice within 2 to 7 days after infection [22]. The authors reported finding significant age-dependent differences in the microglial response due to systemic infection (Figure 1). For instance, in young infected mice compared to control (pyrogen-free sterile isotonic injected saline), there was an increase in microglial cell number in the caudate nucleus as early as 2 days and as late as 7 days after infection. There was also an increase in microglial cell number in the cortex, hippocampus, and thalamus, as well as increased number of CD45- and CD11b-positive cells at 7 days post-infection. In middle-aged mice compared to control/uninfected mice, similar findings were present in the cortex, hippocampus, and thalamus. However, on days 3 and 7, there was moderate microglia activation in the hippocampus and thalamus, as determined by their morphology. Importantly, when comparing young to middle-aged mice, the middle-aged mice presented a higher number of CD45- and CD11b-positive cells at all time points tested when compared to the young infected mice. Additionally, morphological microglial activation was not detected in young infected mice, but there was a moderate activation in the thalamus and hippocampus of middle-aged infected mice. Similarly, mRNA expression of Iba-1 revealed higher levels of Iba-1 at days 3 and 7 in middle-aged infected mice when compared to young infected mice.

In addition, the authors reported an upregulation of mRNA for high mobility group box-1 (HMGB-1) in the brain of young infected mice compared to controls, and a decrease in interleukin (IL)-6 and C1q at day 7. In addition, upon comparing middle-aged infected mice to controls, they found an upregulation of IL-1β and C1q mRNA, and lower levels of IL-6. Most importantly, when comparing young infected mice to middle-aged infected mice, there was an upregulation of mRNA for HMGB-1, tumor necrosis factor (TNF), IL-1β, and C1q within 7 days post-infection in middle-aged mice (Figure 1).

Moreover, an analysis of microglial genes with anti-inflammatory effects revealed a downregulation in the mRNA expression of CXCR1 and CD47 in young infected mice compared to controls, and when comparing middle-aged infected mice to controls, there was an upregulation in the mRNA levels of CX3CR1, CD200R, and CD47. In addition, in an analysis of the Toll-like receptor (TLR) signaling cascade components, there was a downregulation in the mRNA expression of MAPK1 and A20 when comparing young infected mice to controls. Furthermore, in middle-aged infected mice, there was an increase in mRNA expression for TLR-2 and MAPK1 compared to controls, and when comparing young infected mice to middle-aged infected mice, TLR-2 mRNA levels were higher in the middle-aged mice (Figure 1).

Taken together, in the study by Hoogland et al., the authors showed that there was an increase in microglial cell count and a more pro-inflammatory environment (HMGB-1, TNF, IL-1β, and C1q) in middle-aged infected mice [22]. This study is consistent with previous evidence showing that inflammaging, or age-related inflammation, is naturally heightened in the nervous system [32]. Moreover, the authors disproved their hypothesis that anti-inflammatory microglia-specific genes are responsible for the elevated inflammatory response in aged brains since the expression of anti-inflammatory mediators (CX3CL1, CX3CR1, CD200R, and CD47) was elevated in middle-aged brains following infection. Thus, the cause for the increase in pro-inflammatory genes remains to be elucidated. Importantly, the authors used *E. coli* to systemically infect the mice instead of LPS since LPS has been shown to cause a greater neuroinflammatory response than live or heat-killed bacteria, which does not accurately model the clinical scenario. This clinically relevant model of sepsis provides evidence that systemic infection leads to a greater microglial-mediated inflammatory response in the brain due to age; however, the etiology of this heightened immune response mediated by microglia still needs further exploration.

Furthermore, the authors have acknowledged the limitations of their study, one such being that they only used male mice even though there is evidence that the number and phenotype of microglia can differ between females and males [33]. There is also evidence that sex-specific differences exist in the immune response and cell signaling [34]. Additionally, the authors examined the effects of *E. coli* infection, a Gram-negative bacterium. There has been evidence that Gram-positive and Gram-negative infections can have different reactions [35]. Though not significant, Skar et al. showed that there appears to be higher levels of anti-inflammatory cytokines in Gram-positive infections and higher levels of pro-inflammatory cytokines in Gram-negative infections. Thus, it is pertinent to also study the effects of sepsis induced by Gram-positive bacteria to determine if there is a difference in the inflammatory response between Gram-positive and Gram-negative infections in young and middle-aged mice. Lastly, the authors only examined mediators of pathways downstream of the TLR pathway. They suggested that since the data they obtained did not support their hypothesis, a potential reason for the greater inflammatory response seen in middle-aged mice could be due to a cascade of regulators or perhaps a lack of inhibitory regulators in the TLR cascade itself due to the upregulation of TLR-2 and MAPK1 in middle-aged mice. However, it is also possible that there is an upregulation of some inflammatory factors involved downstream of this pathway as well. There are many proteins involved in the TLR cascade and many checkpoints to terminate the cascade if needed. Furthermore, deficiency in the TLR cascade has already been linked to many inflammatory diseases, and TLR inhibitors are promising therapeutic agents [36]. Moreover, there is evidence that many systems work together to regulate each other, including the complement system, autophagy, and the microbiome [37], suggesting that a cascade of regulators may be responsible for increased neuroinflammation in aged mice. Additionally, downstream of TLR signaling is inflammasome activation. There is evidence that the inflammasome plays a role in neuroinflammation seen in sepsis [38], and it has been found to be upregulated in old age [32], also making it a promising candidate for why neuroinflammation is increased in old age. Therefore, further investigation should be done on the TLR cascade and its downstream pathways in order to determine if some aspect of these pathways could be the cause of neuroinflammation due to systemic infection.

Finally, in the study by Hoogland et al., the authors looked at pro-inflammatory and anti-inflammatory mediators of the immune response; however, it is important to consider that microglia have other roles besides the regulation of the inflammatory response, such as synaptic pruning, synaptic transmission, and circuitry plasticity [39]. Thus, future studies could aim to understand the contribution of microglia in the context of aging following infections in these areas that extend beyond inflammation.

In conclusion, the authors argue that the reason for the elevated inflammatory response in the brain is not likely due to a downregulation of anti-inflammatory genes specific to microglia. However, further studies are needed to quantitatively evaluate the levels of pro- and anti-inflammatory proteins produced by microglia in order to identify cut-off points that can be used to determine when the environment is more pro-inflammatory or when it is more anti-inflammatory. Importantly, the model of sepsis used by Hoogland et al. provides evidence that systemic infection leads to a greater microglial-mediated inflammatory response in the brain due to age, and it provides a more clinically relevant model to the study of systemic infection; moreover, the etiology of the heightened immune response mediated by microglia in aging and infections still needs further exploration.

## Figures and Tables

**Figure 1:**
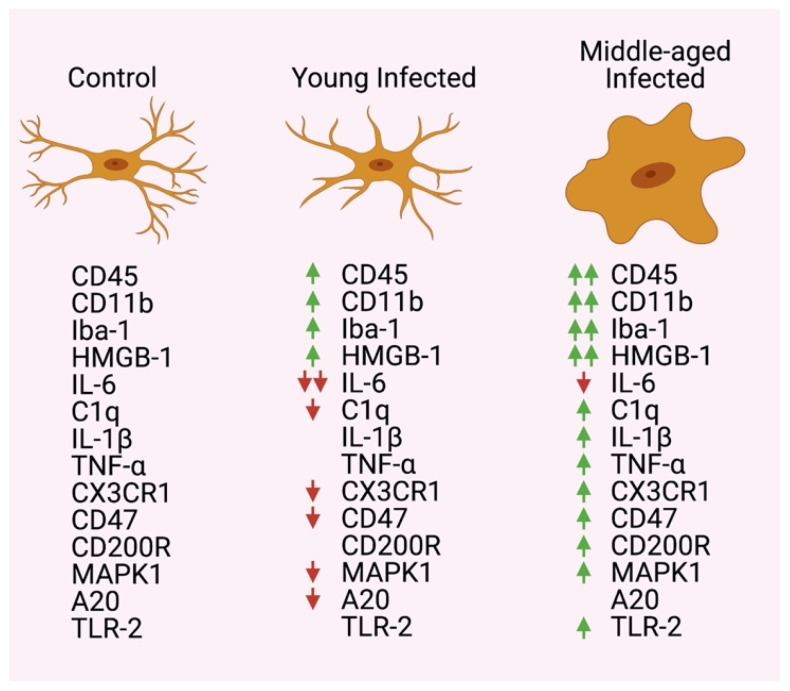
Expression changes between control/uninfected, young infected, and middle-aged infected mice. Two arrows indicate a greater increase or decrease between young and middle-aged infected groups.
